# Evaluating the safety and outcomes of third-trimester selective termination in dichorionic twin pregnancies with discordant anomalies—a standardized approach for counseling

**DOI:** 10.1007/s00404-026-08305-6

**Published:** 2026-01-10

**Authors:** Adeline Walter, Anne Flöck, Jorge Jiménez-Cruz, Brigitte Strizek, Ulrich Gembruch, Annegret Geipel

**Affiliations:** Department of Obstetrics and Prenatal Medicine, University Hospital Bonn, Venusberg-Campus 1, 53127 Bonn, Germany

**Keywords:** Late selective termination, Third trimester selective termination, Ddichorionic twin pregnancy, Discordant anomalies

## Abstract

**Objective:**

To evaluate procedure-related preterm birth (PTB) following third-trimester selective termination (ST) in DC twins and to compare delivery timing with expectantly managed discordant DC twins and non-anomalous DC twins.

**Methods:**

A retrospective cohort study was conducted of all DC twin pregnancies undergoing third-trimester ST (> 28 weeks) at a tertiary care center (2003–2023). Pregnancies were classified as having procedure-related complications (delivery ≤ 4 weeks) or uneventful (delivery > 4 weeks). Comparator cohorts included expectantly managed discordant DC twins and non-anomalous DC twins. Outcomes included timing of delivery, cumulative incidence of PTB, and risk factor analysis.

**Results:**

90 women with DC twin pregnancies elected for ST and 85 procedures were completed. Outcome was available for 81 cases; 48 (59.3%) delivered ≤ 4 weeks after ST and 33 (40.7%) delivered later. Clinical chorioamnionitis was more common within the group delivered ≤ 4 weeks (35.4% vs. 0%; *p* = 0.001). Cumulative PTB incidence showed accelerated delivery between 32 and 34 weeks after ST. Independent risk factors for delivery ≤ 4 weeks included polyhydramnios (OR 5.68) and reduction of the presenting fetus (OR 6.51). Comparator cohorts exhibited substantially lower PTB incidence.

**Conclusion:**

Third-trimester ST in DC twins is associated with high PTB risk, but excellent co-twin survival. The first 4 weeks after ST represent a critical vulnerability period, and risk is strongly influenced by identifiable preprocedural factors. These findings support individualized counseling, later scheduling in high-risk pregnancies, should be considered.

**Supplementary Information:**

The online version contains supplementary material available at 10.1007/s00404-026-08305-6.

## What does this study adds to the clinical work

**Table:** 

- Provides absolute risk estimates for perinatal outcomes after third-trimester selective termination in discordant DC twins.	
- Offers comparative outcomes from DC twin pregnancies with and without discordant malformations not opting for selective termination.	

## Introduction

Dichorionic (DC) twin pregnancies can be complicated by discordant anomalies in 1–2%, with usually only one fetus affected [1, 2]. Having a potential impact on the prognosis of both fetuses, risks of in utero or postnatal demise along with the possible hazard to the healthy co-twin must be evaluated prior to prenatal counseling [1, 3]. In this scenario, selective termination of the affected fetus might be considered as a possible option for parents-to-be, if local legislation permits [1, 4].

Since its introduction in the late 1970s, selective termination in DC pregnancies has been associated with high co-twin survival, particularly when performed early in gestation [5]. Contemporary studies show that procedures before 18 weeks carry the lowest rates of pregnancy loss and extreme preterm birth compared with later interventions [1, 6]. However, some anomalies are not detected until the 18- to 22-week anatomical survey, and diagnosis may be further delayed by extended genetic testing, evolving fetal imaging findings, or parental decision-making [7–10]. In such circumstances, clinicians may *opt* to defer selective termination to the third trimester to avoid the heightened risk of second-trimester pregnancy loss [6]. This approach, however, introduces competing risks—including procedure-related preterm birth (15–30%), increased technical complexity, spontaneous labor, and unintended live birth of the affected fetus [6].

Despite these clinically meaningful trade-offs, the evidence base guiding third-trimester procedures remains sparse. Published cohorts are limited by small sample sizes, wide gestational age ranges (often 18–34 weeks), and incomplete reporting of indications for timing, thereby limiting their applicability to true third-trimester decision-making [6–13].

To address these gaps, we report the largest single-center cohort to date of women with DC twin pregnancies undergoing third-trimester selective termination (> 28 weeks). Our objectives were: (1) to evaluate the association between gestational age at procedure and procedure-related complications, defined as delivery within 4 weeks; and (2) to compare gestational age at birth between three contemporaneous DC twin cohorts—selective termination, expectant management of discordant anomalies, and non-anomalous DC twins—to refine risk estimates and inform evidence-based counseling for third-trimester selective termination.

## Material and methods

### Study design and population

We conducted a retrospective cohort study of all women with DC twin pregnancies discordant for a structural or genetic anomaly that underwent third-trimester selective termination (> 28 + 0 weeks) at the University of Bonn (Germany) between January 2003 and June 2023. Chorionicity was assigned in the first trimester according to standard criteria. All patients received comprehensive anatomical ultrasound, including fetal echocardiography and Doppler assessment, performed by maternal–fetal medicine (MFM) specialists. Counselling included discussion of expectant management and third-trimester selective termination. When third-trimester selective termination was chosen, risks, benefits, and procedural details were reviewed before written informed consent was obtained. Procedures were generally scheduled at 31–32 weeks, hypothesized as a window balancing minimal second-trimester loss with lower rates of spontaneous preterm labor.

### Exposure and group assignment

Patients were retrospectively categorized into:

Group 1 (procedure-related complications): delivery ≤ 4 weeks after selective termination.

Group 2 (uneventful course): delivery > 4 weeks after selective termination.

In parallel, all DC twin pregnancies managed during the same period were classified into three comparison cohorts:

1. Third-trimester selective termination cohort of DC twin pregnancies discordant for anomalies (this study population).
2. Expectantly managed DC twin pregnancies discordant for anomalies.
3. Non-anomalous DC twin pregnancies.

### Procedure

Selective termination was performed percutaneously under transabdominal ultrasound guidance by an intravenous—into the extraabdominal part of the umbilical vein—or an intracardiac injection of 15% potassium chloride, via either a 22 gauge or 20 gauge spinal injection needle. Intracardiac injection was performed when the umbilical cords were not clearly differentiated between the twins or when the umbilical cord was not accessible due to the distance and fetal position, respectively. All procedures were conducted by accredited specialists in maternal–fetal medicine. Fetal asystole was confirmed within 1 min after injection. Daily ultrasound scans were repeated until 48 h after the intervention to confirm fetal well-being of the remaining co-twin, with the majority of these patients being discharged after 2o days. Regular follow-up visits were scheduled at least 2wo weeks after the procedure in general and to monitor fetal growth in the further course. All patients received two doses of betamethasone for fetal pulmonary maturation prior to intervention, unless early-stage delivery occurred after betamethasone had already been administered. All procedures reported in this study were performed in accordance with the applicable legal framework.

### Data collection

Demographic and clinical data were collected by reviewing electronic medical records of each patient and neonate, opting for third-trimester selective termination. Missing data were requested by phone from the parents. Clinical data included pregnancy course/events, e.g., pre-interventional data defining risk factors: cervical length/shortening prior procedure, multiple invasive procedures, prior selective termination (previous amniocentesis/chorionic villus sampling, amnioreduction), preterm contractions, estimated fetal weight of the reduced twin, as well as fetal growth of the healthy co-twin, fetal position of the reduced affected twin (presenting *vs.* non-presenting), gestational age at intervention, and the presence of a polyhydramnios of the affected twin at the time of the procedure. Cervical shortening was defined as a cervical length ≤ 25 mm in the third trimester measured by transvaginal ultrasound examination [14–16]. Polyhydramnios was defined according to the International Society of Ultrasound in Obstetrics and Gynecology (ISUOG) Practice Guidelines as an amniotic fluid index (AFI) ≥ 24 cm or a single deepest vertical pocket (SDP) ≥ 8 cm in the second and third trimesters. Preterm contractions were defined as a patient being hospitalized and admitted to tocolytics. Postprocedural course was assessed by preterm prelabour rupture of membranes (PPROM), clinical chorioamnionitis defined by Triple I (intrauterine inflammation, infection, or both) criteria, and preterm birth (PTB) [17, 18]. Overall neonatal survival was defined as survival at hospital discharge.

### Outcomes

The primary outcome was to examine the effect of gestational age at the time of procedure performed by comparing groups 1 and 2 for significant differences.

The secondary outcome was risk of preterm birth following third-trimester selective termination, analyzed via cumulative incidence curves for the three comparison DC twin cohorts.

### Data analysis

Statistical analysis was performed using the Statistical Package for Social Sciences (SPSS 25.0, SPSS Inc., Chicago, IL, USA) statistical software. Continuous variables are reported as a median, and interquartile range (IQR) as difference between the 75th (Q3) and 25th (Q1) percentiles of the data. Categorical data are expressed as frequencies (n) and percentages (%). Mann–Whitney- U test and Chi-squared test (χ2) were applied for comparison. Univariate binary logistic regression analysis was made to evaluate the predictive value for prior defined adverse outcome parameters. Multiple regression analysis was further performed with group assignment (delivery within 4 weeks vs. > 4 weeks after third-trimester selective termination) as the dependent variable, and polyhydramnios, cervical insufficiency, reduced affected twin (presenting vs. non-presenting), and preterm rupture of membranes as independent variables.

Association was expressed by the odds ratio. A *p* value of < 0.05 was considered significant. Informed consent was obtained from every patient participating in this study for data collection, analysis, and their use for research. As the institutional review board of the University of Bonn does not require formal ethical approval for retrospective archived studies, ethics approval statement is not applicable.

## Results

**Fig. 1:**
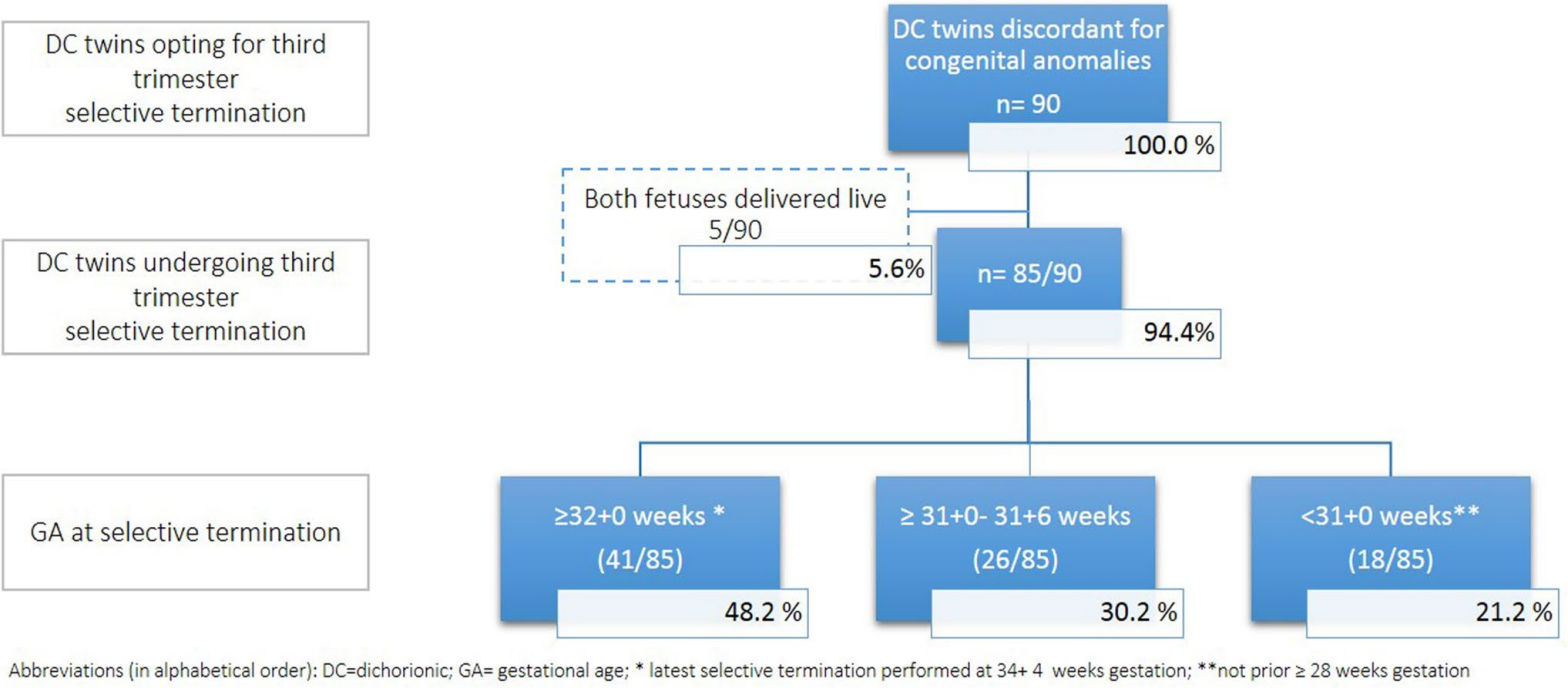
Flowchart displaying the included study population and gestational age at third-trimester selective termination in dichorionic twin pregnancies discordant for congenital anomalies

Between 2003 and 2023, 160 women with dichorionic (DC) twin pregnancies discordant for a congenital anomaly presented to our tertiary referral center. Selective termination in the third trimester was chosen in 90 pregnancies, and the procedure was successfully performed in 85 (94.4%). In five cases (5.6%), selective termination could not be undertaken because of early preterm birth occurring before the scheduled procedure; all five had identifiable risk factors. Pregnancy outcome was unavailable in 4 of the 85 completed cases (4.7%). Figure 1 displays details of inclusions and exclusions of the entire study population.

### Group allocation

Of the 81 pregnancies with available outcome data, 48 (59.3%) delivered within 4 weeks of the procedure (Group 1, procedure-related complications), whereas 33 (40.7%) delivered more than 4 weeks afterward (Group 2, uneventful). Baseline characteristics were similar between groups except for maternal age (*p* = 0.017), as shown in Table 1.

**Table 1:** Baseline maternal characteristics

Characteristics	All *n* = 81*	Group 1 *n* = 48	Group 2 *n* = 33	*p*-value
Maternal age (years)	35.00 (31.5–39.0)	33.5 (28.3–37.0)	36 (32.5–40.0)	0.015
BMI (kg/m^2^)	26.6 (22.4–29.2)	26.1 (22.9–29.1)	26.7 (21.7–30.4)	0.996
Nulliparity (%)	47/81 (58.0)	30/48 (62.5)	17/33 (51.5)	0.363
Mode of conception				
Spontaneous (%) ART (%)	36/81(44.4) 45/81 (55.5)	21/48 (43.8) 27/48 (56.3)	15/33 (45.5) 18/33 (54.5)	0.530 0.511
Frist- and second-trimester diagnostic karyotyping				
CVS (%) AC (%)	9/81 (11.1) 48/81 (59.3)	5/48 (10.4) 28/48 (58.3)	4/33 (12.1) 20/33 (60.6)	0.540 0.511
GA at AC performed	21.0 (18.3–24.0)	21.2 (19.8–24.8)	18.9 (16.8–24.0)	0.262
GA at presentation	20.7 (16.7–23.7)	20.9 (18.1–25.0)	20.3 (16.6–23.0)	0.459

Group 1: defined as delivery within 4 weeks of the procedure; group 2: defined as delivery > 4 weeks after the intervention; continuous variables are presented as median (interquartile range (IQR), Q1–Q3) and categorical variables are presented as number (percentage)

*AC* amniocentesis, *ART* assisted reproductive technology, *BMI* body mass index, *CVS* chorionic villus sampling

*Of the entire study population consisting of *n* = 85 cases, outcome was not available in 4 cases, and assignment in one of the groups was therefore not performed

Indications for selective termination are summarized in Table 2. The most common were chromosomal abnormalities (44.7%), followed by central nervous system anomalies (35.3%). Twenty-one pregnancies had an anomaly diagnosed before 16 weeks; however, selective termination was postponed to the third trimester in 18/21 (85.7%) because of parental hesitation or delay in decision-making, including 13/21 (61.9%) cases of trisomy 21, 1 case with a mosaic of trisomy 18, and further 4 cases with malformations affecting the central nervous system. In the remaining 3/21 (14.3%) cases, ST was postponed due to extended genetic testing.

**Table 2:** Indications for third-trimester selective termination (≥ 28 weeks) in 81 women with dichorionic twin pregnancies discordant for congenital anomalies

Indication	*n* (%) *n* = 81*	Group 1 *n* = 48	Group 2 *n* = 33
Chromosomal defects	37 (45.7)	19	18
Trisomy 21 Others	25 12	9 10	16 2
Central nervous system	26 (32.1)	17	9
Congenital heart defects	4 (4.7)	2	2
Skeletal disorders	6 (7.1)	5	1
Various**	7 (8.2)	5	3

Group 1: defined as delivery within 4 weeks of the procedure; group 2: defined as delivery > 4 weeks after the intervention

*Of the entire study population consisting of *n* = 85 cases, outcome was not available in 4 cases, assignment in one of the groups was therefore not performed

**Various indications, including fetuses with multiple, not further specified abnormalities. Detailed information on indications is provided in the supplementary data (Supplementary Table 1)

The planned timing for selective termination was ≥ 31–32 weeks. In 67/85 pregnancies (78.8%), the procedure occurred as scheduled. In 18/85 cases (21.2%), selective termination was advanced to < 31 weeks because of: progressive cervical shortening 5/18 (27.8%), severe polyhydramnios 5/18 (27.8%), early fetal growth restriction of the unaffected twin with pathological Doppler parameters 3/18 (16.7%), and preterm contractions 2/18 (11.1%). In the remaining three cases (16.7%), the procedure was preponed due to parental anixiety. Procedure-related complications are displayed in Table 3 for both groups. Only the occurrence of clinical chorioamnionitis defined by Triple I criteria occurred significantly more frequently (*p* = 0.001) in group 1. GA at intervention in both groups was similar (31.6 weeks *vs.* 32.0 weeks of gestation; *p* = 0.296). While in group 1, most preterm deliveries occurred between 32 and 34 weeks of gestation, in group 2 most preterm deliveries were orn between 34 and 37 weeks of gestation.

**Table 3:** Complications after third-trimester selective termination, categorized by group 1 (defined as delivery within 4 weeks of the procedure) and group 2 (defined as delivery > 4 weeks after the intervention)

Complication	All *n* = 81*	Group 1 *n* = 48	Group 2 *n* = 33	*p* value
Uterine contractions treated with tocolytics	31 (38.3)	20/48 (41.7)	11/33 (33.3)	0.578
Pathological CTG	4 (4.9)	2/48 (4.2)	2/33 (6.1)	0.615
PPROM after selective termination	11 (13.6)	10/48 (20.8)	1/33 (3.1)	0.023
Clinical chorioamnionitis defined by Triple I [17, 18]	17 (21.00)	17/48 (35.4)	0/33 (0)	< 0.001

*CTG* cardiotocography, *PPROM* preterm prelabour rupture of membranes, Triple I intrauterine inflammation, infection, or both

*In four cases outcome was not available, assignment to one of the groups was therefore not performed

With regard to the entire study population, in 16.0% (13/81) delivery took place within 1 week after the intervention, in 48.1% (39/81) within 2 weeks, in 54.3% (44/81) within 3 weeks, and in 59.3% (48/81) within 4 weeks, thus representing the highest risk of delivery within the first 2 weeks after the intervention.

**Fig. 2:**
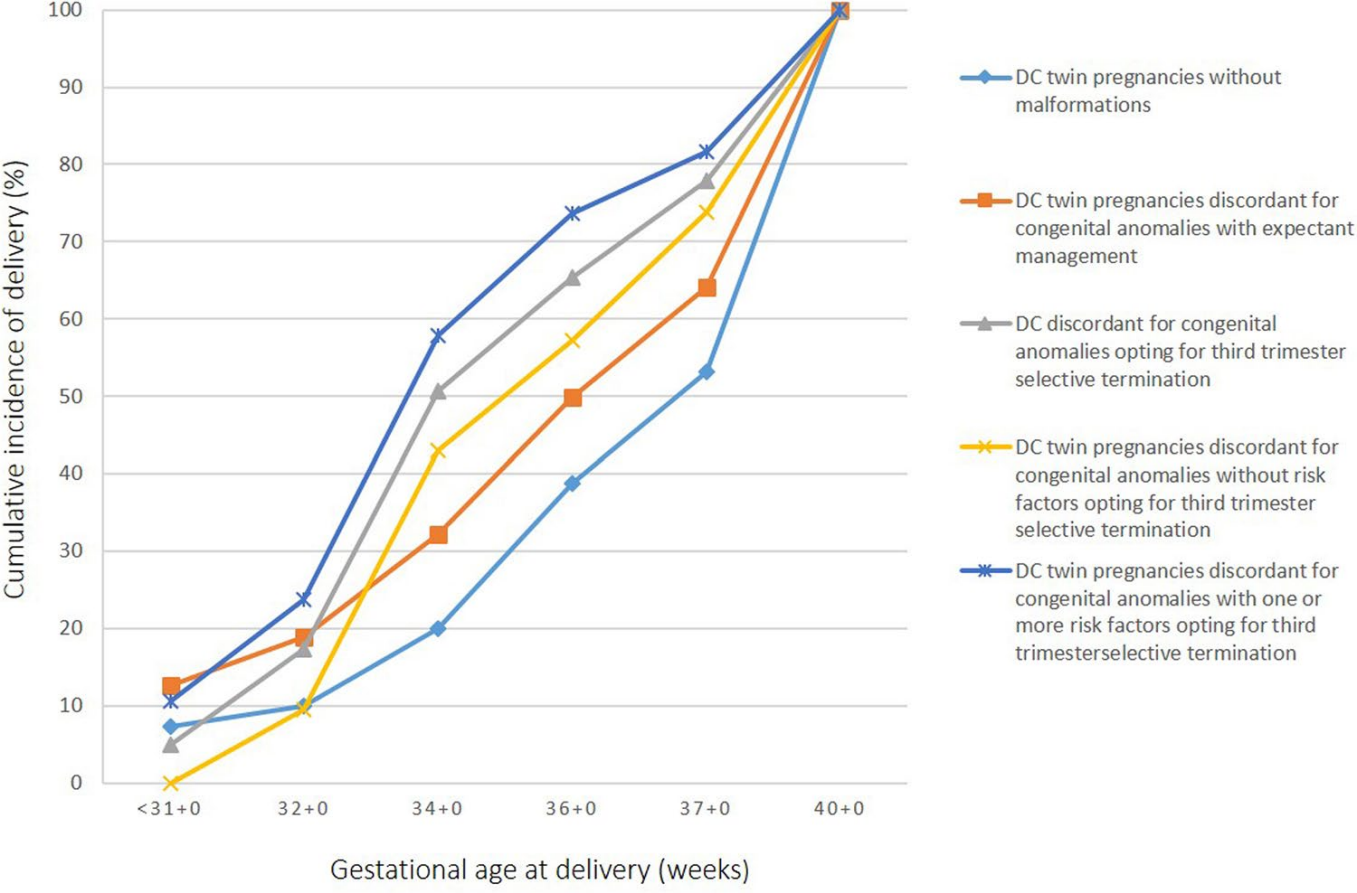
Cumulative incidence of delivery according to gestational age. Data were obtained for cases of pregnancy with dichorionic (DC) twins without malformations (*n* = 2205), DC twins with malformations not opting for selective termination (*n* = 223), and DC twins with discordant anomalies (*n* = 81) opting for third-trimester selective termination presenting to University of Bonn during the period of 003–2023. Additionally, cumulative incidence is shown for DC twin pregnancies undergoing selective termination stratified by the absence (*n* = 42; yellow line) or presence (*n* = 39) of relevant risk factors prior to the procedure. Risk factors were defined as having a polyhydramnios, cervical shortening, or a fetal growth restriction (FGR) of the unaffected twin (light blue line)

Figure 2 illustrates the cumulative incidence of delivery by gestational age, comparing: DC twins without malformations (*n* = 2205), DC twins with anomalies under expectant management (*n* = 223), and DC twins undergoing third-trimester selective termination (*n* = 81), further stratified by presence or absence of predefined risk factors. DC twins without malformations had the lowest PTB incidence, representing the physiological baseline. Expectantly managed discordant DC twins demonstrated similar trajectories over GA, albeit with an overall increased risk of PTB rates through gestation. However in both groups, the PTB rate almost doubled between 32 and 34 weeks of gestation.

Among selective termination cases without identifiable risk factors, PTB incidence at 32 weeks approximated that of non-malformed DC twins (9.5% vs. 10.0%). By 34 weeks—post-intervention—the incidence rose 4.5-fold to 42.9%. Further course of PTB rate was then comparable for both groups (Fig. 2).

Among selective termination cases with risk factors, PTB incidence increased 2.3-fold between and –32 weeks (10.5% → 23.7%) and 2.5-fold between 32 and –34 weeks (23.7% → 57.9%) (Fig. 2).

Univariate analysis of possible prognostic risk factors predicting delivery within 4 weeks are summarized in Table 4. Significant results were identified for polyhydramnios (*p* = 0.006, OR 4.91, 95% CI 1.6–15.2), reduction of the presenting fetus (*p* = 0.001, OR 7.23, 95% CI 2.3–23.4), preterm premature rupture of membranes (*p* = 0.048, OR 6.42 (0.7–53.1), as well as a progressive cervical shortening (*p* = 0.031, OR 4.31, 95% CI 1.1–16.3). On multivariable regression analysis, polyhydramnios (*p* = 0.018, OR 5.68, 95% CI 1.3–23.8) and reduction of the presenting fetus (*p* = 0.010, OR 6.51, 95% CI 1.58–26.86) were the only independent predictors of composite early complications leading to delivery within 4 weeks.

**Table 4:** Univariate analysis of possible risk factors leading to delivery within 4 weeks after third-trimester selective termination

Parameter	Odds ratio (95% CI)	*p*-value
Polyhydramnios	4.91 (1.6–15.2)	0.006
Presenting fetus reduced	7.23 (2.3–23.4)	0.001
Invasive procedure during the entire pregnancy* and than *before invasive procedures including invasive testing (amniocentesis and chorionic villus sampling)	1.48 (0.6–3.6)	0.381
Preterm premature rupture of membranes**	6.42 (0.7–53.1)	0.048
Cervical length ≤ 25 mm	4.31 (1.1–16.3)	0.031
Body mass index [kg/m^2^]	1.02 (0.9–1.2)	0.681
Gestational age at procedure	0.811 (0.55–1.2)	0.295

*Invasive procedures including invasive testing (amniocentesis and chorionic villus sampling), ** occurring after selective termination

## Discussion

In this large single-center cohort, third-trimester selective termination in dichorionic twins was associated with a 100% survival of the unaffected co-twin but carried a substantial risk of procedure-related preterm birth (59.3% within 4 weeks; overall PTB 75.5%). Our findings provide robust evidence clarifying which pregnancies are at highest risk for early delivery after the intervention, thereby enabling more individualized, evidence-based counseling.

Gestational age at the time of the procedure did not differ between the procedure-related complicated (group 1: delivery within 4 weeks) *vs.* uneventful group (group 2: delivery after 4 weeks) (*p* = 0.296), suggesting that timing alone does not drive risk. Comparable results have not been published, as in most cases second trimester selective termination were compared to third-trimester procedures [6–8, 13, 19]. The present study isolates the third trimester and demonstrates that pregnancy-specific risk factors, not procedural timing, seem to determine outcome within this gestational window.

Our cumulative PTB rate (75.5%) is in line with Bennasar et al. (72.7%) [8], but differ from Greenberg and Weissbach et al. (PTB rate of 46.4% and 63.6%), likely reflecting differences in timing (procedures performed earlier in pregnancy; median gestational age of 20.3 weeks in the study by Greenberg et al. compared to 31.9 (IQR, 31.1–32.1) in our cohort) [7], and inclusion of very late procedures (> 33–35 weeks) that carry inherently lower PTB risk [13].

With regard to PTB prior < 28 weeks, < 32 weeks, < 37 weeks, only two studies provided data [8, 13]. Bennasar et al. (selective termination ≥ 24 weeks; reporting on *n* = 22 cases, performed in one case at 24 + 2 week and 21 cases ≥ 28 weeks), calculated PTB rates being as high as 4.5%, 22.7%, and 45.5%, respectively [8], and Weissbach et al. reported on PTB rates of 1.8% before 32 weeks and 63.6% before 37 weeks [13]. While our results of 17.3% for PTB prior < 32 weeks and 48.1% prior < 37 weeks are in line with the study by Bennasar [8], difference with the study of Weissbach et al. might be explained by the inclusion of very late procedures (> 33–35 weeks in nearly 1/5 of their study population), possibly indicating that scheduling third-trimester selective termination even later might be possible [13]. Preterm birth < 28 weeks was not calculated in our cohort, as only pregnancies undergoing invasive procedure > 28 weeks were included. However, as in 5 of 90 cases, selective termination was not performed due to preterm birth of both fetuses, PTB rate < 28 weeks might be assumed to be 5.6% in our cohort. The risk of delivering an anomalous fetus while awaiting for a third-trimester selective termination should be taken into consideration and counseled for.

Importantly, we quantify the “risk acceleration window” following third-trimester selective termination (Fig. 2). The steepest rise in PTB occurs between 32 and 34 weeks, irrespective of risk factor profile (although with the highest increase in the risk-associated group). After 4 weeks postprocedure, PTB trajectories converge with physiological population curves. This pattern supports the conceptualization of a 4-week procedural vulnerability period. Focusing on this 4-week procedural vulnerability period, comparison of PTB rates within group 1: delivery within 4 weeks vs. group 2: delivery after 4 weeks of gestation revealed significant disparities (*p* = 0.001) with 97.9% *vs.* 42.4% in the latter, with further a significant difference in PTB < 32 weeks (27.1% *vs.* 3.1%, *p* = 0.006) and PTB rate prior 34 weeks (77.1% *vs.* 6.1%, *p* = 0.001). As no significant difference in gestational age at the time of the procedure was observed, evaluating potential risk factors or patient characteristics therefore appears to be of highest importance.

Miremberg et al. evaluated pregnancy complication and outcome following selective termination of the presenting *vs.* non-presenting twin and identified selective termination of the presenting twin being associated with higher rates of preterm delivery (75.3% *vs.* 37.6%) [20]. The amount of dead fetoplacental products leading to a tissue maceration was assumed to cause preterm delivery, which was also found as an independent risk factor for postprocedural PTB as well as cervical length less than 35 mm in the study by Weissbach et al. [13, 20]. In our study, similar results were found. Reducing the presenting fetus significantly increased the risk of delivery within the following 4 weeks (*p* = 0.001, OR 7.23, 95% CI 2.25–23.4), although an association to the amount of dead fetoplacental tissue could not be evaluated, as placental masses were not included in the postnatal weighing. The significant parameters investigated in our study were cervical length ≤ 25 mm (*p* = 0.031, OR 4.31, 95% CI 1.1–16.3), preterm premature rupture of membranes (*p* = 0.048, OR 6.42 (0.7–53.1), and polyhydramnios (*p* = 0.006, OR 4.91, 95% CI 1.6–15.2), with the latter being clearly associated with the identified fetal malformation. These factors should be incorporated into preprocedural risk scoring, improving counseling and potentially allowing procedural timing adjustments. In particular, polyhydramnios and reduction of the presenting fetus should be emphasized during preprocedural counseling, as both were identified as independent predictors of delivery within 4 weeks.

Our comparison with contemporaneous DC twin cohorts demonstrates that expectantly managed discordant pregnancies do not show the marked acceleration in preterm birth (PTB) observed after selective termination. This early postprocedural increase in PTB risk should therefore be balanced against the underlying fetal prognosis, maternal preferences, gestational age-dependent neonatal outcomes, and the presence or absence of risk-modifying factors. Notably, PTB trajectories under expectant management closely resemble those of non-malformed DC twins, supporting expectant management as a reasonable option in selected cases—especially for lethal anomalies or anticipated palliative care when no additional risk factors are present—given the continuous improvement in neurodevelopmental outcomes with increasing gestational age.

In contrast, when selective termination is chosen, our findings show that DC twin pregnancies with identifiable risk factors experience the steepest acceleration in PTB between 32 and 37 weeks compared with those without risk factors (Fig. 2). Accordingly, in pregnancies exhibiting such risk factors, selective termination should ideally be postponed. Conversely, in cases without risk factors, selective termination may be safely undertaken between 28 and 32 weeks.

The strength of this study is that it addresses real-life clinical questions of the risk of PTB in performing a third-trimester selective termination and who is at higher risk for PTB following the procedure, in the so far largest cohort reported. With the performed comparison of the cumulative incidence of preterm delivery in relation to gestational age at birth for (1) DC twins without malformation, *vs.* (2) discordant for congenital anomalies not opting for selective termination *vs*. (3) undergoing selective termination at a single center we provided counseling strategies and management recommendations with a more robust evidence base.

Nevertheless our study has some limitations. First, as no standardized protocol was used, clinically evaluated data were not available from all patients. Moreover, risk factors were only systematically assessed in dichorionic (DC) twin pregnancies that underwent selective termination. As a result, a direct comparison of identified risk factors with DC twin pregnancies affected by malformations but managed expectantly was not performed. The retrospective study design with its common weaknesses and the short follow-up are additional limitations, which must be taken into account, as well as the single-center setting, which may limit generalizability.

In conclusion, third-trimester selective termination in dichorionic twins is a safe procedure regarding co-twin survival, but carries a high risk of preterm birth, particularly within the first 4 weeks after intervention. Risk is driven less by gestational age at the time of the procedure and more by specific preprocedural factors, including polyhydramnios, cervical shortening, multiple invasive procedures, and reduction of the presenting fetus. These findings support individualized counselling and suggest that later scheduling should be considered in high-risk pregnancies. When no risk factors are present, expectant management may remain a viable alternative. Evaluation should be repeated throughout the pregnancy, as risk factors may develop.

## Supplementary Information

Below is the link to the electronic supplementary material.

Supplementary file1 (JPG 84 KB). Kaplan–Meier curve illustrating the cumulative incidence of delivery (%) over time following third-trimester selective termination, stratified by Group 1 (delivery ≤ 4 weeks after the procedure; red curve) and Group 2 (delivery > 4 weeks after the intervention; blue curve)

Supplementary file2 (DOCX 16 KB)

## Data Availability

The datasets are available from the corresponding author on reasonable request.
