# Supplementary figures and images for: Evaluating the safety and outcomes of third-trimester selective termination in dichorionic twin pregnancies with discordant anomalies—a standardized approach for counseling
Source: Arch Gynecol Obstet. 2026 Jan 10;313(1):26. doi: 10.1007/s00404-026-08305-6 (PMC12789172; doi:10.1007/s00404-026-08305-6)

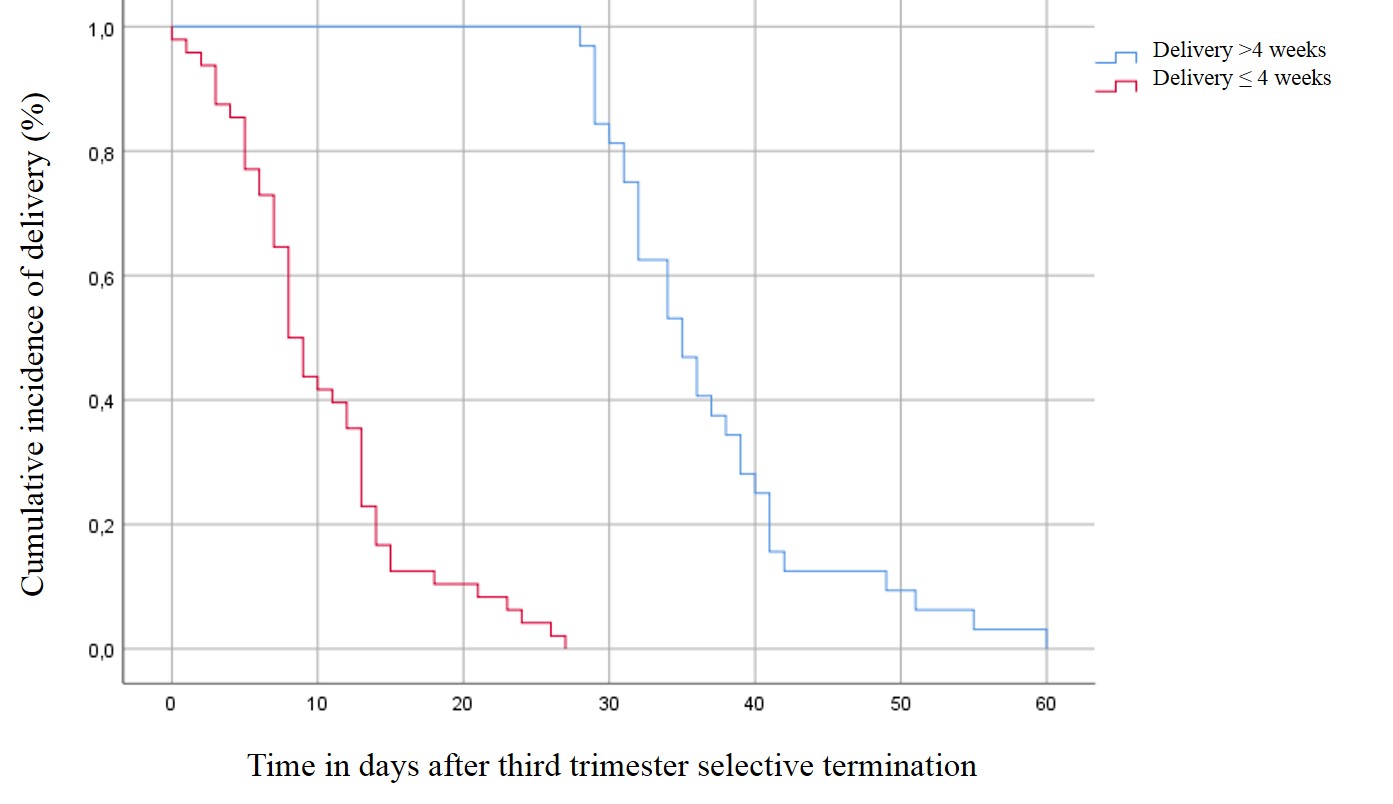

Supplement: Supplementary file 1 — Supplementary file1 (JPG 84 KB). Kaplan–Meier curve illustrating the cumulative incidence of delivery (%) over time following third-trimester selective termination, stratified by Group 1 (delivery ≤ 4 weeks after the procedure; red curve) and Group 2 (delivery > 4 weeks after the intervention; blue curve) [file 404_2026_8305_MOESM1_ESM.jpg]
