# Supplementary material for: Evaluating the safety and outcomes of third-trimester selective termination in dichorionic twin pregnancies with discordant anomalies—a standardized approach for counseling
Source: Arch Gynecol Obstet. 2026 Jan 10;313(1):26. doi: 10.1007/s00404-026-08305-6 (PMC12789172; doi:10.1007/s00404-026-08305-6)
Supplement: Supplementary file 2 — Supplementary file2 (DOCX 16 KB) [file 404_2026_8305_MOESM2_ESM.docx]

**Table 2.** Indications for third trimester selective termination (≥ 28 weeks) in all 85 dichorionic twin pregnancies.

| **Indication** | | n (%)  n=85* | Group 1  n=48 | Group 2  n=33 |
| --- | --- | --- | --- | --- |
| Chromosomal defects | | 38 (44.7) | 19 | 18 |
|  | Trisomy 21  Trisomy 13 and 18  Others | 26  4  8 | 9  3  7 | 16  1  1 |
| Central nervous system | | 30 (35.3) | 17 | 9 |
|  | Malformation of cortical developmental  Holoprosencephaly  Cerebellar abnormalities  Open spina bifida  Hydrocephalus internus  Encephalocele  Intracranial hemorrhage  Agenesis of corpus callosum | 3  1  4  8  5  4  1  4 | 3  1  2  6  3  1  0  1 | 0  0  0  2  2  2  1  2 |
| Congenital heart disease | | 4 (4.7) | 2 | 2 |
|  | Complex TOF  Hypoplastic left heart syndrome | 3  1 | 2  0 | 1  1 |
| Skeletal disorders | | 6 (7.1) | 5 | 1 |
|  | Thanatophoric dysplasia  Skeletal disorders, others  Arthrogryposis multiplex  Fetal akinesia deformation sequence | 2  1  1  2 | 2  1  0  2 | 0  0  1  0 |
| Various | | 7 (8.2) | 5 | 3 |
|  | Omphalocele-exstrophy-imperforate anus-spinal defect complex  Urogenital malformations  Others** | 1  1  5 | 0  1  4 | 1  0  2 |

*In 4 cases outcome was not available, assignment in one of the both group was therefore not performed. Indication were however assessed;**Others including fetuses with multiple abnormalities not further specified. Abbreviations (in alphabetical order): TOF= Tetralogy of Fallot;
